# First-Principle Study of AlCoCrFeNi High-Entropy Alloys

**DOI:** 10.3390/nano16010020

**Published:** 2025-12-23

**Authors:** Andi Huang, Yilong Liu, Jinghao Huang, Jingang Liu, Shiping Yang

**Affiliations:** 1 School of Mechanical Engineering and Mechanics, Xiangtan University, Xiangtan 411105, China; andy_h_china@foxmail.com (A.H.); jingangl2025@163.com (J.L.); 2 School of Aeronautical Engineering, Hunan Automotive Engineering Vocational University, Zhuzhou 412001, China; liuyilong@stu.usc.edu.cn; 3 School of Mechanical Engineering, University of South China, Hengyang 421001, China; huangjh2019@126.com

**Keywords:** AlCoCrFeNi high-entropy alloys, first-principles calculations, phase stability, electronic structure, mechanical properties

## Abstract

AlCoCrFeNi high-entropy alloys (HEAs) are promising materials due to their exceptional mechanical properties and thermal stability. This study employs first-principles calculations based on density functional theory (DFT) to investigate the phase stability and electronic properties of AlCoCrFeNi HEA. The atomic size difference (*δ*) was determined to be 5.44%, while the mixing enthalpy (Δ*H*_mix_) was found to be −14.24 kJ/mol, and the valence electron concentration (VEC) was measured at 7.2, indicating a dual-phase structure consisting of the BCC and B2 phases. The formation energies indicated that the BCC phase exhibits the highest stability under typical conditions. The elastic properties were assessed, revealing Young’s modulus of 250 GPa, a shear modulus of 100 GPa, and a bulk modulus of 169 GPa, which suggest high stiffness. The alloy demonstrated a Poisson’s ratio of 0.25 and a G/B ratio of 0.59, indicating relatively brittle behavior. Microhardness simulations predicted a value of 604 HV0.2, which closely aligns with experimental measurements of 602 HV0.2 at 1300 W laser power, 532 HV0.2 at 1450 W, and 544 HV0.2 at 1600 W. The electronic structure analysis revealed metallic behavior, with the d-orbitals of Co, Fe, and Ni contributing significantly to the electronic states near the Fermi level. These findings offer valuable insights into the phase behavior and mechanical properties of AlCoCrFeNi HEA, which are crucial for the design of high-performance materials suitable for extreme engineering applications.

## 1. Introduction

High-entropy alloys (HEAs) represent a unique class of materials characterized by the mixing of five or more elements in nearly equal atomic proportions. This multi-element composition results in a significant increase in configurational entropy, which promotes the formation of simple solid solutions, such as body-centered cubic (BCC), face-centered cubic (FCC), or hexagonal close-packed (HCP) structures, rather than complex intermetallic phases [1,2]. The four principal effects—lattice distortion, sluggish diffusion, the cocktail effect, and slow coarsening—contribute to the remarkable properties of HEAs, including high strength, hardness, excellent wear resistance, high-temperature oxidation and corrosion resistance, and exceptional thermal stability [3,4,5]. These outstanding characteristics position HEAs as promising candidates for advanced engineering applications, encompassing aerospace [6,7], biomedical devices [8], and catalysis [9].

Among the various HEA systems, AlCoCrFeNi HEAs have garnered significant attention due to their balanced mechanical and chemical properties. The AlCoCrFeNi HEA can exhibit dual-phase (FCC + BCC) or multiphase structures, which are influenced by its composition and processing conditions, subsequently affecting its hardness, ductility, and corrosion resistance [10,11,12]. This unique combination of properties positions it as a strong candidate for high-temperature structural components, such as turbine blades and heat exchangers. However, the coexistence of ordered B2 phases with disordered FCC or BCC matrices presents challenges in controlling phase stability and mechanical performance [13].

To enhance the durability of materials exposed to extreme environments, the development of high-performance coatings has become essential. Extensive research has been conducted on conventional coatings, including ceramic (e.g., Al_2_O_3_ [14], TiSiN [15], CrN [16]), metallic (e.g., Al [17], Cr [18]), and alloy coatings (e.g., Fe-Al [19], Fe-Cr-Al [20]), to improve their high-temperature oxidation and wear resistance. Recently, HEA coatings have garnered increasing attention due to their unique combination of strengthening mechanisms, which provide exceptional mechanical strength, thermal stability, and wear resistance under extreme conditions [21,22,23].

Despite these advantages, the multi-component nature of HEAs introduces complexity to their microstructure, complicating the prediction of their stability and the regulation of their properties [24,25]. Since the macroscopic performance of materials is fundamentally determined by their microstructure and electronic states, a thorough understanding of their atomic-scale behavior is essential. In particular, the stability and oxidation resistance of HEAs are closely linked to their electronic structure. Metals exhibiting higher Fermi levels are more susceptible to electron loss and oxidation, while grain boundaries and defects characterized by low electron density frequently serve as sites for anodic dissolution [26].

First-principles calculations, particularly density functional theory (DFT), have proven to be invaluable tools for predicting material properties such as phase stability, elastic constants, and electronic structures directly from atomic configurations [27,28,29]. These methods have been extensively applied in HEA research to elucidate the mechanisms underlying their stability and mechanical behavior [30,31,32]. For instance, Min Chul Oh et al. [33] identified that atomic size differences, mixing enthalpy, and valence electron concentration (VEC) are critical factors influencing phase formation in AlCoCrFeNi HEA. Similarly, Zhou et al. [34] employed DFT to predict elastic constants and established the mechanical property balance in Ti-X alloys.

Although previous density functional theory (DFT) studies have investigated the AlCoCrFeNi system [33,35], most relied on separate single-phase models (calculating FCC and BCC independently) and estimated alloy properties using mixing rules. In this study, to address these limitations, first-principles methods were employed to construct an explicit Dual-Phase SQS supercell (containing 200 atoms) alongside standard BCC and FCC models. This approach allows for the direct simulation of the cooperative mechanical response and electronic interactions at the FCC-BCC interface, distinct from standard single-phase approximations. Furthermore, unlike studies validating simulations against equilibrium cast samples, this work couples theoretical predictions with Laser-Directed Energy Deposition (LDED) experiments. This combined framework enables the investigation of how non-equilibrium processing parameters (specifically laser power) induce deviations from theoretical ‘ideal solution’ limits, thereby elucidating the critical role of B2 ordering. Phase stability was evaluated based on formation energies and compared with experimental X-ray diffraction (XRD) data, while atomic-scale structural optimization explored lattice distortion effects. Finally, the band structure, density of states (DOS), and charge density distribution were analyzed to elucidate the electronic origins of stability and high-temperature resistance. These findings are anticipated to provide valuable insights for the design of high-performance HEA coatings for use in extreme engineering applications.

## 2. Materials and Methods

### 2.1. Theoretical Methods and Calculation Models

**Figure 1:**
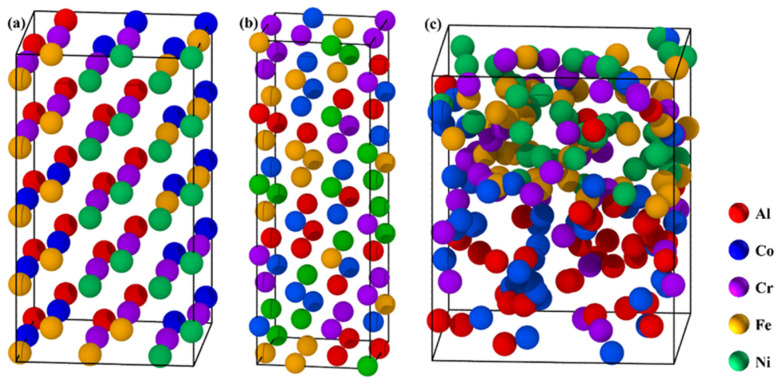
SQS supercell model. (**a**) BCC AlCoCrFeNi of 3 × 3 × 5; (**b**) FCC AlCoCrFeNi of 2 × 2 × 5; (**c**) Biphasic AlCoCrFeNi of xy = 3 × 3/2 × 2z = 5.

The FCC, BCC, and dual-phase models of AlCoCrFeNi HEA were constructed using the special quasi-random structure (SQS) method within the framework of density functional theory (DFT). These models were specifically designed to simulate chemical disorder and local distortions for an equimolar composition, with each element constituting 20 at.%. The GGA-PBE functional and PAW pseudopotentials were utilized for electronic structure calculations. The supercell structures were generated in POSCAR format as follows: Figure 1a depicts the BCC model with 80 atoms in a 3 × 3 × 5 conventional cell, with an initial lattice parameter a = 3.5 Å; Figure 1b illustrates the FCC model comprising 90 atoms in a 2 × 2 × 5 conventional cell, with an initial lattice parameter a = 3.0 Å; Figure 1c presents the dual-phase model consisting of 200 atoms, wherein the BCC and FCC phases are mixed in a 50:50 ratio along the z-direction, with dimensions of xy = 10 Å and z = 12 Å. Monte Carlo atomic substitution (seed 42, 10k–15k iterations) was employed to minimize the Warren-Cowley short-range order (SRO) parameters, which ranged from 0.0035 to 0.052 on average. The BFGS optimization method was applied, utilizing a plane-wave cutoff energy (E_cut) of 450–500 eV, k-meshes varying from 5 × 5 × 6 to 8 × 8 × 4 (with an 8 × 8 × 3 mesh designated for elastic calculations), and SCF Pulay density mixing. The convergence criteria were established as follows: total energy ≤ 1 × 10^−5^ eV/atom, maximum force < 0.03 eV/Å, atomic displacement < 0.001 Å, and stress < 0.05 GPa, with a maximum of 100 optimization steps. The elastic properties were calculated using the stress–strain method, with strains ranging from ±0.5% to ±2%.

### 2.2. Experimental Details

The bulk AlCoCrFeNi HEA samples were prepared using laser-directed energy deposition (LDED). The LDED system comprises a laser generator with a maximum power of 6 kW at a wavelength of 1075 nm, a four-axis coaxial powder feeding laser cladding head, an ABB six-axis robotic arm, a CNC worktable, and protective gas (argon gas with 99.95% purity). The ABB six-axis robotic arm guided the laser cladding head to perform unidirectional scanning in both directions, executing multi-pass and multi-layer deposition to fabricate the bulk AlCoCrFeNi HEA samples. The LDED process parameters were established as follows: laser powers of 1300 W, 1450 W, and 1600 W; a scanning speed of 480 mm/min; a powder feeding rate of 4 r/min; a laser diameter of 3 mm; a defocus of 0 mm; a protective gas flow rate of 10 L/min; an overlap rate of 50%; and a single-layer thickness of 0.8 mm. After deposition, the bulk AlCoCrFeNi HEA was cut into small squares measuring 10 mm × 10 mm × 10 mm using wire cutting for X-ray diffraction (XRD) testing. The phase composition of the deposited AlCoCrFeNi HEA was characterized by XRD (Panalytical, Empyren, Japanese Neo-Confucianism MiniFlex600, Tokyo, Japan) using Cu Kα radiation. The diffractometer was operated at 30 mA and 40 kV, with the diffraction angle (2θ) scanned from 20° to 90° at a scan rate of 5°/min and a step size of 0.02°.

Microhardness measurements were performed using an HVS-1000 Vickers hardness tester (Shanghai Caikang Optical Instrument Co., Ltd., Shanghai, China) with a test load of 200 gf and a hold time of 10 s. Measurements were conducted at intervals of 100 μm perpendicular to the substrate, starting at 50 μm below the surface of the cladding layer.

## 3. Results

### 3.1. Phase Structure

The potential for establishing a set of empirical guidelines to preliminarily predict the formation of high-entropy alloys is frequently investigated, with atomic size difference (*δ*) and mixing enthalpy (Δ*H*_mix_) identified as two of the most critical factors. The commonly employed calculations and their physical meanings are as follows [36]:

(1) $$\delta =\sqrt{\sum_{i=1}^{n}c_{i}{\left(1-\frac{r_{i}}{\bar{r}}\right)}^{2}}\;,\bar{r}=\sum_{i}c_{i}r_{i}$$

where $c_{i}$ is the atomic percentage of the i-th element, $r_{i}$ is its atomic radius, and $\bar{r}$ is the average atomic radius. A smaller *δ* indicates less lattice distortion, which favors the formation of a homogeneous solid solution by allowing different elements to substitute for each other more easily. In contrast, a larger *δ* introduces significant lattice strain, which tends to promote phase separation or the formation of compound phases.

Mixing enthalpy (Δ*H*_mix_) reflects the strength and nature of the chemical interactions between elements. Empirically, the mixing enthalpy of binary pairs is commonly used for combined calculations [37]:

(2) $$\Delta H_{\mathrm{mix}}=\sum_{i<j}4\Delta H_{ij}^{\mathrm{mix}}c_{i}c_{j}$$

Δ*H*_mix_ represents the molar mixing enthalpy of the element *i-j* binary system, $c_{i}$ or $c_{j}$ is the atomic percentage of the i-th or j-th element. When the overall Δ*H*_mix_ is moderate (neither strongly negative nor strongly positive), it favors the formation of a disordered solid solution. A highly negative Δ*H*_mix_ generally promotes the formation of ordered compounds or intermetallic phases, while a strongly positive value tends to lead to phase separation. When the atomic size difference is less than 6.6% [38] and the mixing enthalpy lies between −15 kJ/mol and 5 kJ/mol, it is expected that a solid solution high-entropy alloy will form [39].

Another parameter is the valence electron concentration (VEC), which has been demonstrated to be instrumental in determining the type of lattice structure [40]. According to the literature [39], when the average VEC of an alloy exceeds 8, the system tends to stabilize in an FCC phase. Conversely, when the VEC falls below 6.87, the alloy is more likely to stabilize in a BCC phase. For VEC values ranging from 6.87 to 8, the alloy typically exhibits a coexistence of FCC and BCC structures. The definition of VEC is as follows:

(3) $$VEC=\sum_{i=1}^{n}c_{i}e_{i}$$

where $e_{i}$ is the number of valence electrons, $c_{i}$ is the atomic percentage of the i-th element, and *n* is the number of components.

Table 1 presents the calculated values of atomic size difference, mixing enthalpy, and valence electron concentration for the AlCoCrFeNi HEA. It is evident from Table 1 that the atomic size difference (*δ* = 5.44%) is smaller than the critical value of 6.6%. This indicates that the size difference among the alloying elements is relatively small, which allows the system to maintain a stable lattice structure and satisfies the fundamental conditions for solid solution formation. From a thermodynamic perspective, the mixing enthalpy (Δ*H*_mix_ = −14.24 kJ/mol) is negative, suggesting a strong chemical affinity among the elements. Although this value remains within the empirically accepted range, it approaches the lower limit, indicating that not only is the formation of a solid solution feasible, but there is also a significant driving force for B2 ordering or the precipitation of local intermetallic compounds, particularly involving strongly bonded atomic pairs such as Al–Ni and Al–Cr. Finally, from the standpoint of electronic structure, the VEC of 7.2 falls within the transitional range of 6.87–8.0, which corresponds to the coexistence zone of FCC and BCC phases. This suggests a preference for the BCC phase as the primary structure, while also allowing for the potential coexistence of FCC and B2 ordered phases. Therefore, the AlCoCrFeNi HEA meets the structural stability criteria necessary for HEA formation. However, due to the negative mixing enthalpy, its microstructure is more likely to exhibit dual-phase or even multiphase characteristics, featuring a BCC matrix with B2 ordered phases.

**Figure 2:**
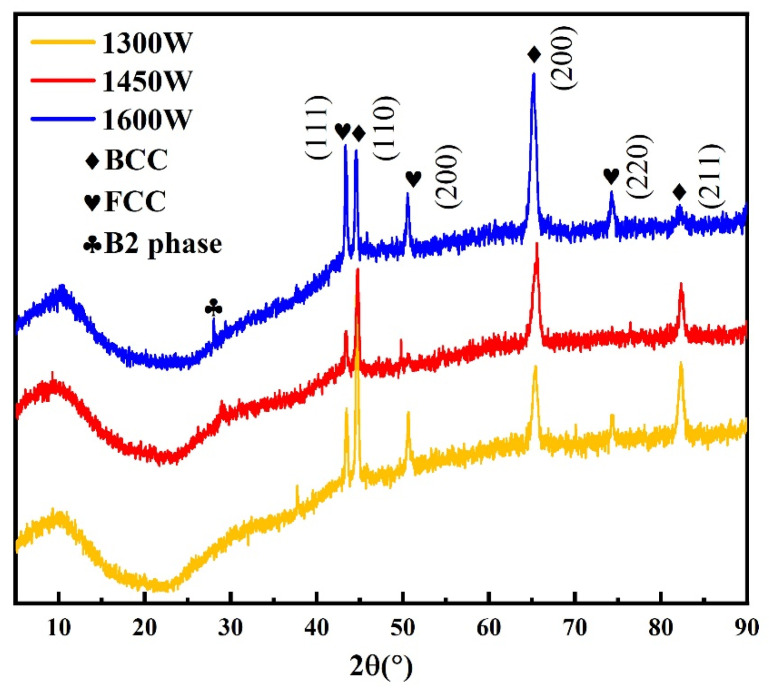
XRD patterns of bulk AlCoCrFeNi HEA prepared under different laser power conditions.

Figure 2 illustrates the XRD patterns of bulk AlCoCrFeNi HEA prepared under different laser power conditions. The XRD characterization results indicate significant phase evolution of the AlCoCrFeNi HEA with varying laser powers. Under the 1300 W laser power condition, the diffraction peaks are broad and exhibit additional superlattice reflections, suggesting that insufficient energy input and slower cooling rates lead to substantial ordering and the formation of the B2 (ordered BCC) phase. At 1450 W, the dominant phase is identified as the BCC solid solution, characterized by strong and sharp diffraction peaks, indicating good crystallinity and a relatively stable solid solution structure. At 1600 W, both enhanced FCC peaks and residual B2 peaks are observed, signifying that the higher energy input modifies the composition and solidification kinetics (including local evaporation, chemical segregation, and rapid solidification capture), ultimately resulting in a complex microstructure with coexisting BCC, FCC, and B2 phases. Overall, the experimental results align with the thermodynamic and electronic structure trends indicated by previously obtained values of *δ* = 5.44%, Δ*H*_mix_ = −14.24 kJ/mol, and VEC = 7.20, demonstrating that the alloy resides in the coexistence zone of FCC and BCC phases and possesses a clear driving force for ordering.

### 3.2. Elastic Properties

In materials with a typical crystal structure, the elastic constants serve as key indicators of the macroscopic mechanical properties under static loading. These constants not only quantify the material’s response to applied stress and strain but also elucidate the fundamental bonding characteristics within the crystal at a microscopic level [41]. For the Biphasic AlCoCrFeNi HEA model, the elastic constants (C_ij_) were computed based on the supercell structure, with the resulting values presented in Table 2. In a cubic crystal structure, characterized by identical bond lengths and angles, only three independent elastic constants exist: C_11_, C_12_, and C_44_. These constants are crucial for determining the material’s stiffness and deformation behavior. Additionally, the mechanical stability of the alloy can be assessed using the mechanical stability criterion specific to cubic crystals, which ensures the material’s ability to withstand applied stresses without experiencing structural instability [42].

According to the Born rule [43], the mechanical stability of a material is determined by the critical condition described in Formula (4). Based on the calculated data, it can be concluded that the Biphasic AlCoCrFeNi HEA exhibits mechanical stability under a stress of 0 GPa. This finding indicates that the alloy remains in a stable state with minimal deformation under low external stress. Additionally, the Cauchy pressure (C_12_–C_44_) serves as a crucial indicator of the material’s plasticity. A positive Cauchy pressure suggests that the material possesses ductility, meaning it can deform without fracturing. In contrast, a negative value would indicate a lack of ductility and increased brittleness [44]. For the Biphasic AlCoCrFeNi HEA, the positive Cauchy pressure signifies significant ductility, which correlates with enhanced toughness and a greater capacity to absorb energy before failure.

(4) $$C_{11}>0,C_{44}>0,C_{11}-C_{12}>0,C_{11}+2C_{12}>0$$

On the other hand, Poisson’s ratio, bulk modulus and Young’s modulus can be further calculated by using the C_ij_, and the calculation formulas are as follows (5)–(10) [45]:

(5) $$B=\frac{C_{11}+2C_{12}}{3}$$

(6) $$E=\frac{9\mathrm{GB}}{3B+G}$$

(7) $$\nu =\frac{3B-2G}{2(3B+G)}$$

(8) $$G_{R}=\frac{5\left(C_{11}-C_{12}\right)C_{44}}{4C_{44}+3\left(C_{11}-C_{12}\right)}$$

(9) $$G_{V}=\frac{C_{11}{-C}_{12}+3C_{44}}{5}$$

(10) $$G=\frac{G_{V}+G_{B}}{2}$$

Table 3 presents the simulated mechanical properties of the Biphasic AlCoCrFeNi HEA, including Young’s modulus (E), shear modulus (G), bulk modulus (B), Poisson’s ratio (ν), and the G/B ratio. The alloy exhibits Young’s modulus of 250.0 GPa, a shear modulus of 100.0 GPa, and a bulk modulus of 169.0 GPa, indicating that it is a relatively stiff material. The Poisson’s ratio is 0.25, slightly below the threshold value of 0.3, suggesting that the material may exhibit more brittle than ductile behavior. Furthermore, the G/B ratio is 0.59, which exceeds the critical value of 0.57, further supporting the conclusion that the alloy demonstrates brittle behavior. These findings indicate that the Biphasic AlCoCrFeNi HEA, characterized by its relatively low Poisson’s ratio and high G/B ratio, is likely to be brittle rather than ductile.

Hardness is a crucial indicator of the overall mechanical properties of materials. To investigate the effect of phase composition on the mechanical properties of the Biphasic AlCoCrFeNi HEA, we calculated the hardness. Typically, the microhardness (HV) of alloys can be predicted from the bulk modulus and shear modulus, as outlined in Formula (11).

(11) $$HV=\left(2\times {\left(\frac{G^{3}}{B^{2}}\right)}^{0.585}\right)-3$$

**Figure 3:**
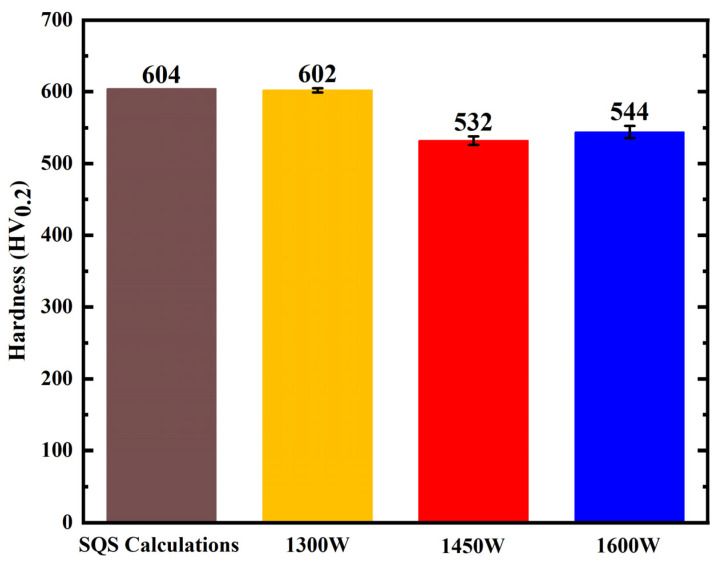
The calculation and experimental microhardness of AlCoCrFeNi HEA.

The microhardness of the AlCoCrFeNi HEA under varying laser power conditions is illustrated in Figure 3. The predicted hardness values, derived from the special quasi-random supercell (SQS) method, are 604 HV0.2, which is slightly higher than the experimental values. Under experimental conditions, the hardness measurements are 602 HV0.2 at 1300 W, 532 HV0.2 at 1450 W, and 544 HV0.2 at 1600 W. These results indicate that hardness decreases with increasing laser power, with a partial recovery observed at higher power levels. As depicted in Figure 2, XRD analysis reveals corresponding phase changes with increasing laser power: the relative intensity of the BCC phase diminishes, while the B2 phase becomes more pronounced. The transformation to the B2 phase likely accounts for the observed decrease in hardness, as B2 structures typically exhibit lower hardness compared to BCC or FCC phases. Consequently, the shift in phase composition with higher laser power plays a significant role in the softening of the material. The C-based predictions closely align with the experimental data, thereby validating the accuracy of this computational approach in predicting the mechanical properties of high-entropy alloys.

### 3.3. Electronic Structure

**Figure 4:**
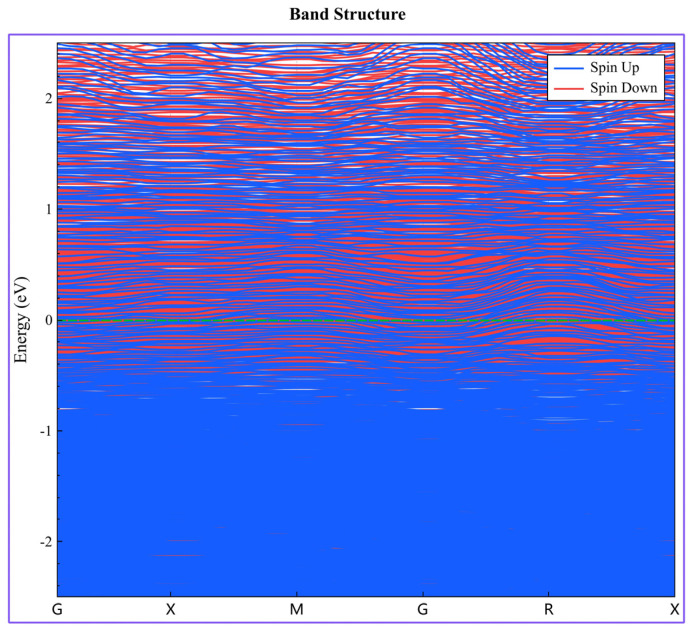
Energy band structure of AlCoCrFeNi HEA.

Figure 4 illustrates the energy band structure of the AlCoCrFeNi HEA along the highly symmetric directions of the Brillouin zone. The green dotted line in the figure indicates the position of the Fermi energy level. The energy band structure of the AlCoCrFeNi HEA reveals that the Fermi level intersects multiple energy bands, confirming the absence of a band gap and indicating typical metallic behavior with high electrical conductivity. The continuous distribution of energy bands across high-symmetry points in the Brillouin zone reflects the free mobility of electrons, further supporting the alloy’s metallic nature. At the Fermi level, the electronic states are primarily influenced by the d-orbitals of Fe, Co, and Ni, which contribute significantly to metallic bonding and conduction, while the s–p orbitals of Al, although contributing less directly to conductivity, play a crucial role in regulating the electronic structure and stabilizing the overall phase. This electronic configuration not only elucidates the strong metallic bonding of the system but also supports the experimentally observed coexistence of the BCC, the FCC, and B2 phases, as the high density of states near the Fermi level provides the driving force for structural competition and ordering. Therefore, the energy band structure analysis demonstrates that the AlCoCrFeNi HEA inherently possesses strong metallic characteristics and complex phase stability, consistent with thermodynamic and experimental observations.

**Figure 5:**
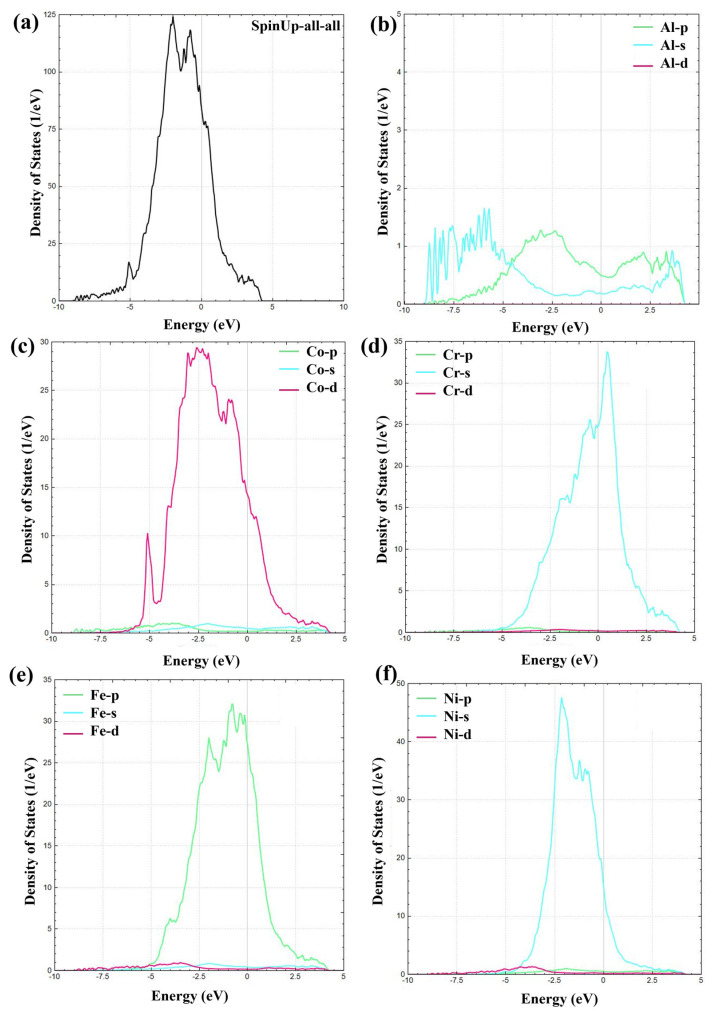
The total and partial density of states of AlCoCrFeNi HEA: (**a**) AlCoCrFeNi HEA; (**b**) Al; (**c**) Co; (**d**) Cr; (**e**) Fe; (**f**) Ni.

The electronic density of states (DOS) of the AlCoCrFeNi HEA, as shown in Figure 5a, confirms its metallic character. The total DOS at the Fermi level (EF = 0 eV) remains finite, indicating the absence of an electronic band gap and the presence of itinerant carriers [46]. A pronounced peak between approximately −4 and −2 eV corresponds to strongly occupied bonding states, while the residual states at EF are primarily derived from the transition-metal d orbitals. Specifically, Co (Figure 5c), Fe (Figure 5e), and Ni (Figure 5f) exhibit broad d-band distributions in the range of −5 to −1 eV, extending across the Fermi energy and thus serving as the dominant contributors to metallic bonding and electrical conductivity [47]. In contrast, Cr (Figure 5d) presents a relatively sharper and more localized d-band peak near −2 to −1 eV, indicative of enhanced d-electron localization or short-range chemical ordering effects compared to Co, Fe, and Ni. Aluminum (Figure 5b) primarily contributes via s-p states at energies below EF, with significantly weaker intensity than the transition metals, implying that Al stabilizes the electronic structure mainly through s-p-d hybridization rather than directly supplying conduction states. The observed overlap between Al s-p and transition-metal d states across −6 to 0 eV suggests strong orbital hybridization, which plays a crucial role in bond formation and phase stability [48]. Moreover, the large DOS at EF derived from multiple d orbitals not only strengthens metallic bonding but also increases the likelihood of electronic instabilities, such as ordering transitions, magnetic polarization, and multiphase coexistence, which aligns with experimental observations of BCC/FCC/B2 structures in this alloy system.

**Figure 6:**
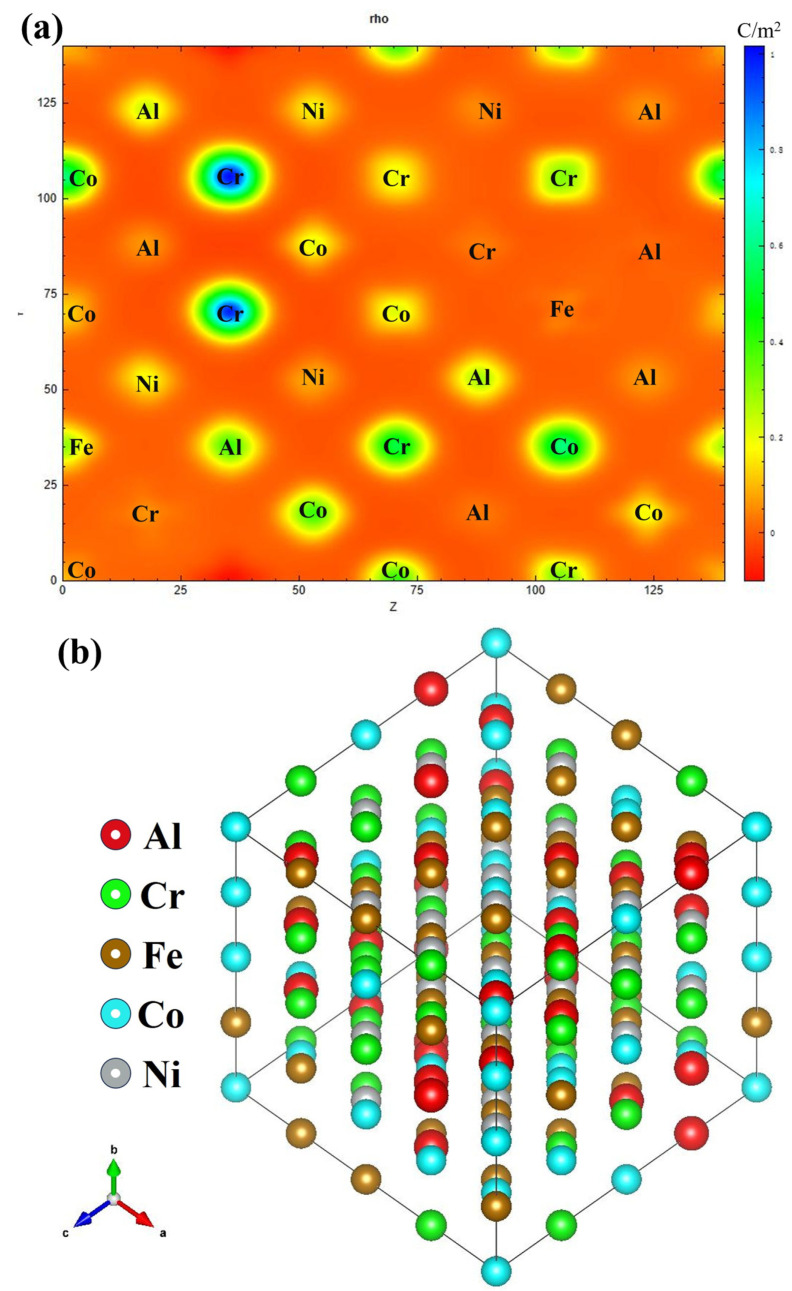
(**a**) The charge density, (**b**) the charge density difference diagram of AlCoCrFeNi HEA along crystal plane (110).

Figure 6a illustrates the total charge density distribution of the AlCoCrFeNi HEA projected onto the (110) plane. High-density regions are observed around the transition metal sites (Cr, Co, Fe, Ni), while a relatively weak density is noted around the Al atoms, reflecting their s–p contribution. Clear electron-density bridges between the transition metals and Al atoms indicate a strong s–p–d hybridization. Figure 6b presents the charge density difference map on the same (110) plane, as outlined in Formula (12), defined as follows:

(12) $$\Delta \rho =\rho_{\mathrm{alloy}}-\sum_{i}\rho_{i}^{\mathrm{atom}}$$

where red (positive) regions denote charge accumulation and blue (negative) regions represent charge depletion. Contour levels are typically set at ±0.01 e/Å^3^ to highlight bonding and depletion features.

## 4. Conclusions

In this study, first-principles calculations were employed to investigate the phase stability, mechanical properties, and electronic structure of the AlCoCrFeNi HEA. The results reveal that the alloy predominantly exhibits a dual-phase structure comprising BCC and B2 phases, with BCC being the more stable phase under typical conditions. Additionally, the calculated mechanical properties and electronic structure provide insights into the material’s strength, stiffness, and metallic nature. These findings can guide the design of high-performance AlCoCrFeNi HEAs for applications in extreme environments, such as aerospace and high-temperature engineering components. The following conclusions can be drawn:

1. AlCoCrFeNi HEA tends to form a dual-phase structure, with the BCC phase being more stable than the FCC phase, as indicated by the formation energy calculations and supported by experimental XRD results.
2. The atomic size difference (*δ*) of 5.44%, negative mixing enthalpy (ΔHmix) of −14.24 kJ/mol, and valence electron concentration (VEC) of 7.2 suggest a preference for dual-phase formation, primarily BCC, with strong thermodynamic driving forces for phase stability.
3. The calculated mechanical properties show Young’s modulus of 250 GPa, a shear modulus of 100 GPa, and a bulk modulus of 169 GPa, indicating high stiffness. The Poisson’s ratio of 0.25 and G/B ratio of 0.59 suggest the material is relatively brittle.
4. Experimental microhardness measurements (602 HV0.2 at 1300 W, 532 HV0.2 at 1450 W, and 544 HV0.2 at 1600 W) show good agreement with theoretical predictions, validating the accuracy of the computational approach.
5. The electronic structure confirms the metallic nature of AlCoCrFeNi HEAs, with significant contributions from transition metal d-orbitals near the Fermi level, indicating strong metallic bonding and high electrical conductivity.

## Figures and Tables

**Table 1:** Atomic size difference, mixing enthalpy and valence electron concentration of AlCoCrFeNi HEA.

Material Type	*δ* (%)	Δ*H*_mix_ (kJ/mol)
AlCoCrFeNi HEA	5.44	−14.24

**Table 2:** Elastic constants of the Biphasic AlCoCrFeNi HEA.

HEA	Elastic Constants C_ij_ (GPa)		
	C_11_	C_12_	C_44_
AlCoCrFeNi	232.3	137.4	135.3

**Table 3:** Young’s modulus (E/GPa), Shear modulus (G/GPa), Bulk modulus (B/GPa), Poisson’s ratio (ν) and G/B values of the Biphasic AlCoCrFeNi HEA.

HEA	E	G	B	ν	G/B
AlCoCrFeNi	250.0	100.0	169.0	0.25	0.59

## Data Availability

The original contributions presented in this study are included in the article. Further inquiries can be directed to the corresponding author.
